# Conceptualising the public health role of actors operating outside of formal health systems: The case of social enterprise

**DOI:** 10.1016/j.socscimed.2016.11.009

**Published:** 2017-01

**Authors:** Michael J. Roy, Rachel Baker, Susan Kerr

**Affiliations:** a Yunus Centre for Social Business and Health, Glasgow Caledonian University, Glasgow, UK; b School of Health and Life Sciences, Glasgow Caledonian University, Glasgow, UK; c Glasgow School *for* Business and Society, Glasgow Caledonian University, Glasgow, UK

**Keywords:** UK, Social enterprise, Public health, Conceptual modelling, Logic models

## Abstract

This paper focuses on the role of actors that operate outside formal health systems, but nevertheless have a vital, if often under-recognised, role in supporting public health. The specific example used is the ‘social enterprise’, an organisation that seeks, through trading, to maximise social returns, rather than the distribution of profits to shareholders or owners. In this paper we advance empirical and theoretical understanding of the causal pathways at work in social enterprises, by considering them as a particularly complex form of public health ‘intervention’. Data were generated through qualitative, in depth, semi-structured interviews and a focus group discussion, with a purposive, maximum variation sample of social enterprise practitioners (n = 13) in an urban setting in the west of Scotland. A method of analysis inspired by critical realism – Causation Coding – enabled the identification of a range of explanatory mechanisms and potential pathways of causation between engagement in social enterprise-led activity and various outcomes, which have been grouped into physical health, mental health and social determinants. The findings then informed the construction of an empirically-informed conceptual model to act as a platform upon which to develop a future research agenda. The results of this work are considered to not only encourage a broader and more imaginative consideration of what actually constitutes a public health intervention, but also reinforces arguments that actors within the Third Sector have an important role to play in addressing contemporary and future public health challenges.

## Highlights

• Considers the health role of actors outside of formal health systems.
• Focuses upon the example of social enterprise.
• Presents empirically-informed conceptual model of causal pathways.
• Encourages a more imaginative consideration of what we think of as an ‘intervention’.

## 1 Introduction

There is an increasing recognition that political, economic, social, cultural, environmental, behavioural and biological factors can all favour or harm health (Marmot and Wilkinson, 2006, Whitehead et al., 2001, Whitehead and Dahlgren, 2007). Outside of the obvious role that governments can play in addressing many of the social determinants of health (Bambra, 2013, McQueen et al., 2012, Wismar et al., 2013) there is also growing recognition of the public health role played by actors who are less obvious, operating outside of formal healthcare systems. Organisations and groups within civil society (Giarelli et al., 2014) often aim, implicitly or explicitly, to tackle aspects of social vulnerability (Galea et al., 2005) that we understand to be critical to health, but their work – at least in public health terms – can often go unrecognised, or at best under-recognised, by health service funders, researchers and policymakers. Furthermore, the actors *themselves* may not recognise the impact of their work in public health terms, since “their influence on health [is, rather,] a product of their primary intent” (Hanlon et al., 2012, p. 169).

The ‘third sector’ is conceptualised as the space in which private forms of individual or collective action are undertaken for wider public benefit, rather than for personal financial enrichment (Evers and Laville, 2004, Salamon and Sokolowski, 2016), roughly equivalent to what the UK Department of Health has recently referred to as the Voluntary, Community and Social Enterprise (VCSE) sector (see VCSE Sector et al., 2016). The role of the third sector in delivering various policy outcomes has been increasing in prominence since at least the advent of New Public Management (Alcock and Kendall, 2011) and social enterprise – the part of the third sector engaged in trading – has been used in particular by successive governments, first coming to policymakers’ attention in the late 1990s as a mechanism to regenerate deprived communities (Blackburn and Ram, 2006; Teasdale, 2012) but then as part of Third Way-inspired policies emblematic of New Labour (Haugh and Kitson, 2007). Over time, social enterprise has become a preferred vehicle for delivering a range of public services, most notably in health and social care (see Roy et al., 2013 for a full discussion). Thus, when social enterprise is discussed in relation to public health, it is most often in connection with its role or potential as a mechanism for *delivering* health and social care services, either as an alternative or complement to mainstream public provision (Hazenberg and Hall, 2014) or as a mechanism for enhancing community involvement in service design, particularly in rural contexts (Farmer et al., 2012, Muñoz, 2011).

Recently a small body of literature has started to emerge which considers the health and/or well-being impact of social enterprise-led activity as a ‘product of their primary intent’, ranging from grey literature written by practitioners (Boswell et al., 2009, McDermid et al., 2008, Westwater, 2009) to theoretical or conceptual papers written by academics (Donaldson et al., 2011, Farmer et al., 2016, Muñoz et al., 2015), to the point that there are now a small number of systematic reviews. Roy et al. (2014) presented some, albeit limited, evidence that social enterprise-led activity can impact positively on mental health, self-reliance/esteem and health behaviours, reduce stigmatization and build social capital, all of which can contribute to overall health and well-being. Most of the studies found in that review focused upon the outcomes of Work Integration Social Enterprises (WISEs), a form of social enterprise designed to provide a supportive environment for vulnerable people, such as those with severe mental or physical disabilities, operating “along with other businesses, within the larger economic structure, but with a view to creating workplace structures that are inclusive and enabling of worker productivity and well-being” (Krupa and Chen, 2013, p. 197). The recent systematic review by Mason et al. (2015, p. ii116) takes a broader perspective, assessing social enterprise on its ability to address health equity within the wider context of ‘social innovation’. Although they find inconsistent evaluative evidence of impacts, some of the benefits they identify include “the mobilization of latent or unrealised value through new combinations of (social, cultural and material) resources; growing bridging social capital and purposeful approaches to linking individual knowledge and experience to institutional change”.

Social enterprises will often use a variety of methods to report and/or measure their impact, whether at their own behest or due to the requirements of funders, commissioners, or other stakeholders. However, we do not yet fully possess the tools to effectively evaluate the (health) outcomes of social enterprise-led activity. An important gap in evidence remains in relation to empirical studies which explicitly frame social enterprise as a complex form of public health ‘intervention’, and which employ methods aimed specifically at unpacking and assessing how the intervention might work, the identification of ‘active ingredients’, and how they are exerting their effect (viz. Craig et al., 2008). This paper aims to contribute towards filling that gap; to explore how social enterprise practitioners – whether implicitly or explicitly, deliberately or otherwise – conceptualise the impact of their activities upon the health and well-being of the individuals and communities they seek to support. We focus specifically upon community-led, and owned, social enterprises working outside of formal health systems, working to address one or more aspects of social vulnerability within the communities they serve. We develop and present an empirically informed conceptual model of causal pathways linking the activities of social enterprises, intermediate outcomes that result from those activities, and ultimately their impact on health and wellbeing, with a view to providing a framework for a future research agenda.

## 2 Methodology and methods

The study was undertaken in Glasgow, a city in west central Scotland, which has some of the poorest health in western Europe and a health profile more in common with eastern European countries (McCartney et al., 2012). Glasgow also has a well-developed social enterprise sector: a recent report (GSEN and Social Value Lab, 2013) describes social enterprise in Glasgow as having ‘scale as well as substance’ and estimates some 509 social enterprises operating within the city, with a combined turnover of £767m and employing over 10,000 people. An interest of this study was to provide evidence of variety in order to make sense of the heterogeneity of experiences and practices of different social enterprises and contexts in Glasgow, which would then support the process of theory building, and the development of a suitably robust conceptual model. Thus, a purposive (Mason, 1996) maximum variation (Given, 2008) sample of social enterprises based in the city was identified. A phased approach (Miles and Huberman, 1994) to both sampling and data collection was undertaken, with the principle of generating *enough* data to inform the construction of a plausible and theoretically coherent conceptual model. Whether data saturation ever truly occurs is a longstanding debate. However, Morse's (1995, p. 148) advice was followed; that “researchers cease data collection when they have enough data to build a comprehensive and convincing theory.” The social enterprises were identified using a combination of a dataset provided by a Glasgow-based enterprise support agency and then by drawing upon contacts and personal knowledge of the sector. Ethical approval was sought and approved by the appropriate committee within the university. Overall, data were gathered from 13 different social enterprise practitioners between October 2013 and February 2015. We use the term ‘social enterprise practitioners’ as a catch-all term to describe those working in social enterprises. Not all of the people we spoke to were the leaders or founders of the organisation: we deliberately sought people to take part who had both sufficient operational knowledge and day to day familiarity with the people they support. In smaller organisations this was often the leader, but in larger organisations the most appropriate person was often found several rungs down the management ladder.

The name of each organisation has been anonymised, disguised using a codename ranging from ‘Alpha’ through to ‘Mike’, although we also explained to participants that it may be possible for someone with sufficient knowledge of the social enterprise sector in Scotland to recognise the organisations from the descriptions herein. The name of the respondent was disguised in alignment with the codename given to the social enterprise, so the name of the social enterprise practitioner based at Alpha has been called Alan (a male respondent), while Christine (a female) is based at the social enterprise we have assigned the code Charlie. While the sample was purposive and the aim was not to generalise, it is worth stating that five of the 13 social enterprises examined were found to be in the most deprived areas in the whole of Scotland, and so broadly consistent with an observation that 42% of Glasgow-based social enterprises are located in Scotland's most deprived neighbourhoods (GSEN and Social Value Lab, 2013). Seven of the social enterprises stated that impacting upon health and/or well-being was a component of their social mission, while the remaining six did not explicitly mention “health” and/or “well-being” in their social mission, which was established from reading publicly available company documents. Data from these documents were used to support the process of developing key criteria in order to construct the sample. The sample of social enterprises was then constructed based upon maximum variation on these criteria, including: the industrial sector in which they operate; their geographical reach; turnover; length of time in operation; and number of employees. The sample is described in Table 1, with each organisation's social mission distilled to provide the brief description of each organisation.

Where the research aim is to address causal mechanisms and to move beyond surface appearances to explore the processes involved, it is appropriate to study individuals in context (Kazi, 2003, Sayer, 2000) and so in-depth, one-to-one semi-structured interviews were undertaken, based on a topic guide. After the interviews the interim findings, including an early iteration of the model, were discussed at a focus group of social enterprise practitioners. The focus group was convened somewhat opportunistically; the lead author was responsible for suggesting social enterprise participants for another project, and used that opportunity, after gaining suitable permissions, to present the draft model to some people who had previously been interviewed and some who had not, with a view to improving validity of the model and also the possibility of generating new perspectives not previously considered. Both the interviews and focus group were recorded and transcribed ‘intelligent verbatim’.

With the goal to locate, extract and infer causal pathways within the data, a method of analysis inspired by the critical realist philosophy, Causation Coding (Miles et al., 2014, Saldaña, 2013), was employed. To support this process, once satisfied that each transcript was an accurate representation of the interview, each was imported into the computer assisted qualitative data analysis programme QSR NVivo. Critical realism involves employing ‘abductive’ inference (Peirce, 1932, Timmermans and Tavory, 2012), which involves moving from the level of observations and lived experience to examine the underlying structures and mechanisms that account for the phenomena involved (McEvoy and Richards, 2006). The Causation Coding method allows identification of the mental models that participants use to explain events and their causes through identifying the ‘processual links’ embedded within discourses, through language that incorporates chronological processes (words such as first, initial, then, next, future, and so on) to provide a sense of ‘storylined’ influences and effects. Although narratives were not always linear in terms of a ‘story’ from specific causes to outcomes, coding of the data in this way helped the development of sequences reflecting three general “temporal categories” (Miles et al., 2014, p. 242): namely: *antecedent variables* (the baseline conditions before the action or changes); *mediating variables* (those events, states, processes, and/or factors that initiate changes or action of some kind); and *outcomes* (the consequent results of antecedent and mediating variables). Organising in such a way helped build a “logical chain of evidence” (Miles et al., 2014, p. 242) to support the construction of the conceptual model, and the rigour of the links in this chain – the possible paths of causation and causal mechanisms – have been strengthened through “moving backward and forward among empirical data, research literature, and emergent theory” (Dey and Teasdale, 2013, p. 255) in an abductive, iterative manner known as ‘systematic combining’ (Dubois and Gadde, 2002).

## 3 Findings

### 3.1 Antecedent variables

The antecedent variables represent the conditions before the action or changes, and so can be understood to be consistent with various aspects of social vulnerability that social enterprises exist to address, which are often multi-faceted, complex and highly context dependent. Given the high levels of deprivation faced by most of the communities in which the social enterprises are based, it is perhaps unsurprising that most of them mentioned that they had a particular focus upon addressing aspects of environmental deprivation or, more accurately, the social consequences that were seen as being linked to this. For example, one aspect of deprivation that was mentioned several times (particularly by Bravo and Alpha) was in relation to poor quality housing:

“Although it has improved over the years, there is a considerable amount of poor housing, you know damp housing, poor housing” (Alan).

Several of the organisations (particularly Alpha, Delta, Echo and Kilo) reported focusing upon supporting older people suffering from isolation within their communities. Alan, for example, spoke of the loneliness of elderly people who have just come out of hospital, perhaps whose families have moved away, and both Doreen and Karen explained that this can give rise to particular health concerns. Similarly, Edward spoke of the work that Echo undertook with older people who were (in his words) “socially isolated”, perhaps because of a diagnosis of dementia. Edward explained that social isolation and exclusion can have a detrimental impact upon mental health, which can then cause physical health to deteriorate. While isolation and marginalisation of older people was identified as a key problem faced by most of the organisations, several of the social enterprises (particularly Bravo, Hotel, Foxtrot and Golf) reported supporting the specific needs of younger people, who are often classed as vulnerable because they live chaotic lives. Ian, for example, spoke of the young people that India supports being on the “margins of the community” with issues often exacerbated by involvement with addictions, or a range of other multiple complex issues:

“… people who are coming from very chaotic situations. It might be long term addictions, whatever that addiction might be, or just coming out of very chaotic family lifestyles or lots of terms in and out at Her Majesty's pleasure for whatever reason” (Bill)

Issues relating to ethnicity, particularly in relation to refugee or asylum status, were raised by a few of the respondents (Bravo, Charlie and India) while a number of the social enterprises (Alpha, Bravo, Charlie, Delta, Hotel and Juliet) considered that lack of employment was a particular problem in their area, with several detrimental impacts related to this, both as cause and as effect. Homelessness and poor health, for example, were both recognised as states that mutually reinforce each other, contributing to unemployment and isolation and marginalisation from society. Harry spoke of the importance of his organisation providing an element of structure, continuity and support for people “as they went up and down on the rollercoaster of dealing with their alcoholism” (Harry).

### 3.2 The forms of ‘intervention’

In order to address the various aspects of social vulnerability briefly outlined above, each of the social enterprises were found to employ one of four different types of ‘intervention’: two of the social enterprises (Alpha and Echo) were involved in providing personal care services; two others (Foxtrot and Golf) used the arts and creativity as the principle vehicle by which they supported individuals. Another two (Hotel and India) were involved in work integration of people marginalised or distanced from the labour market i.e. they were Work Integration Social Enterprises (WISEs) the specific form of social enterprise discussed earlier. The remaining seven (Bravo, Charlie, Delta, Juliet, Kilo, Lima and Mike) were involved in various forms of community development.

### 3.3 Mediating variables: *what* the social enterprises do, and *how*

The mediating variables represent the events, states, processes, and/or factors that initiate changes or action of some kind (Saldaña, 2013). If we accept that an intervention is a “set of actions with a coherent objective to bring about change or produce identifiable outcomes” (Rychetnik et al., 2002, p. 119) then the social enterprises were found to ‘intervene’ in seven different ways or ‘sets of actions’: *providing meaningful work*; *engendering a supportive and safe environment*; *improving knowledge and skills*; *expanding social networks*, *building trust and co-operation*; *improving access to information and welfare*; *improving public awareness and understanding of social issues;* and*, building feelings of self-worth and value to society*. Each category of mediating variable is explained and discussed in turn.

#### 3.3.1 Providing meaningful work

Several of the social enterprises (Hotel, India and Juliet) explained that they provided work for people traditionally excluded or distanced from the labour market. Thought was given to the *type* and *nature* of work provided, with the intention to provide ‘good work’. ‘Good’ not because it was highly paid (in fact, in all of the cases it was not) but rather because the work was seen as ‘meaningful’. Meaningful work was provided by the various social enterprises in several different ways. India, for example, focused upon the level of pay and working environment and conditions: they are a ‘living wage’ employer and talked of promoting ‘worker dignity’ in many different ways, including investing time and effort in getting to know well the young people they support, many of whom come from troubled or chaotic backgrounds.

Juliet, on the other hand, did not, at least as a matter of course, offer remunerative employment to the people her organisation supported, but undertakes quite a complex process to match volunteering opportunities to potential volunteers. Harry, meanwhile, spoke of investing time and energy into training the volunteers and paid workers at Hotel in traditional craft skills.

#### 3.3.2 Engendering a safe and supportive environment

Two of the social enterprises (Hotel and India) spoke, specifically, about the necessity of creating a safe place where people can feel that they are valued and protected. Hotel provides an alternative to mainstream employment for people recovering from various addictions, training people to create wooden objects from old reclaimed wood for onward sale, predominantly for use in gardens: so sheds, raised beds, garden benches, and so on. Harry mentioned that any number of the participants “fall off the wagon” on occasion, but the organisation acts as a “non-judgemental” anchor point for people to return to when they feel able. He operates a strict no-alcohol or drugs policy, not least for safety reasons, and will send people home if they turn up to work or volunteer under the influence. Harry describes the organisation as a ‘lifeboat’ and ‘safe haven’ for people trying to get their lives back on track.

Like Hotel, India is also a Work Integration Social Enterprise and in the 25 years it has been in existence has diversified into a range of services aimed at supporting vulnerable young people, including carer centres, housing support services, community care and a number of café and food outlets. The organisation provides a range of training, work experience, guidance and support opportunities, and great care is taken to provide a safe and supportive environment. They provide training, mentorship and encouragement to help broaden and develop the participant's role and responsibilities, and all staff have access to an independent counselling service if, for any reason, they feel they cannot approach their line manager with a particular problem. Ian explained that character references are provided for young people if they have to attend court. Even if they have to go to prison, Ian described occasions when jobs have been kept open for young people for after they are released. This was a similar philosophy to Hotel: Ian felt that it was important for the young people to know that even if the rest of their life was going ‘off the rails’, one aspect of their lives – the social enterprise and the people who support them – could be constant and be relied upon.

#### 3.3.3 Improving knowledge and skills

Several of the social enterprises (Bravo, Charlie, Delta, Foxtrot, Hotel and Juliet) explained that they worked to improve the knowledge and skills of the people they support. Most of the social enterprises provided education and learning programmes of various forms, particularly with a view to supporting those distanced from, or disadvantaged in, the labour market. Doreen, for example, spoke of the Adult Literacy and Numeracy classes that they provide, and that two of their volunteers provide support for a drop-in facility to teach people to become proficient with basic IT skills. Jill spoke of providing training to one of her volunteers to become a qualified adult literacy tutor in order that she could then support local people to read and write.

Harry, meanwhile, spoke of the degree of skill in woodworking that some of the people that his organisation supports have developed as a result of working at the organisation. Christine, meanwhile, explained her role in developing the skills of street magazine vendors in order that they may succeed in their role. However, not everyone spoke of improving knowledge and skills for the sake of employability. Fiona, for example, trains young people in a variety of circus skills:

“she is a fantastic juggler, she can do spinning plates, she can spin fire, she can do some club juggling and she has been working on climbing so not just the skill building but thinking about how those skills can be put into more of a performance and linking them together with climbing skills as well.” (Fiona)

Bill, meanwhile, spoke of providing a room for a local Tamil group that meets to teach their children the Tamil language as a means of supporting people to celebrate their heritage and preserve their distinct cultural identity.

#### 3.3.4 Expanding social networks, building trust and co-operation

All of the social enterprises interviewed one-to-one (that is, Alpha, Bravo, Charlie, Delta, Echo, Foxtrot, Golf, Hotel, India, Juliet and Kilo) explained that bringing people together, helping people to expand their social networks and make friends was a key part of their work, and this was done in several different ways. Both Alan and Edward, for example, spoke of the personal services they provide to vulnerable people, many of whom were isolated because of ill health and/or disability. Both took great store in ensuring there was an appropriate level of quality and length of time devoted to human contact, and suggested that this approach has a much greater benefit upon the people being supported. Both Bravo and Juliet bring people together in a variety of different ways, and act as a catalyst for local people to bring their own ideas forward to promote connections between individuals and different groups. Bill, for instance, spoke of the ways in which he worked to bring together people from different races or cultural backgrounds.

#### 3.3.5 Improving access to information and welfare

Five of the social enterprises (Bravo, Delta, India, Juliet, and Kilo) specifically mentioned that one of their key roles was to provide people with information, particularly in relation to benefit entitlements, and thus improve access to welfare. Bravo, for instance, provides a drop-in advice service, employing an advice worker to provide both advocacy and advice. Similarly, Delta works with a local money advice centre to provide information to people who are looking to come off benefits and into employment, and specific information aimed at young people.

#### 3.3.6 Improving public awareness and understanding of social issues

Both Bravo and Charlie were specifically involved in raising awareness with the general public on social issues. One idea the practitioners at Charlie had to raise awareness of homelessness was to encourage local Members of Parliament to become a street vendor for a day. This idea later evolved into encouraging prominent business people to also try their hand at selling street papers. The work of Bravo, in particular, to improve public awareness of the plight of asylum seekers and refugees overlaps significantly with the final mediating variable.

#### 3.3.7 Building feelings of self-worth and value to society

Bravo became the focal point of a campaign of resistance against inhumane treatment of asylum seekers and refugees back in the mid-2000s. After the UK Government decided unilaterally to reduce the asylum population by fifty percent, a number of dawn raids by the police started to happen. Ordinary people with, on any measure, very little power to stand up to the police or the Government of the day, took the decision to stand up for a group of people with even less power, simply because they railed at what they perceived as unjust treatment of a group of people they considered part of their community. They employed tactics of civil disobedience which, it was claimed, led to the “shaming” of the Government, and eventually influenced a change in policy. Such changes have undoubtedly had a profound effect upon the lives of many people as a result, including upon those who took part in the activities, whose self-worth improved and were even recognised by the national press and received several awards as a result of this work. One slight note of caution in relation to this particular example is that it is exceptional, particularly when compared with the other cases where focus is rather more upon the ‘everyday’ reality of the work of social enterprises. Ash Amin (2009, p. 47) however, memorably describes the work of social enterprise practitioners as ‘extraordinarily ordinary’:

“It is extraordinary because its values and motivations are neither reducible to, nor commensurate with, those that prevail in the market economy or in bureaucratic organisations. It is ordinary because its people, goods, services, work practices and achievements are unglamorous. But this ordinariness also draws on the exceptional effort of individuals and organisations working in the most testing circumstances to meet social needs and empower communities.”

The next section turns to an analysis of the outcomes that working in such ‘extraordinarily ordinary’ ways can have on the health and well-being of individuals and communities.

### 3.4 Outcomes

The outcomes are the product of the mediating variables acting upon the antecedent variables and are categorised into *physical health*, *mental health* and *social determinants*. These categories are interrelated and can often impact upon, and reinforce and/or confound, each other.

#### 3.4.1 Physical health

The types of outcomes that have been grouped under physical health include: improved nutrition; improved health behaviours, decrease in illicit or dangerous behaviours; and improved physical well-being and healing. Each outcome is discussed in turn.

Improved nutrition was one perceived outcome of Alpha's work to provide healthy, balanced and affordable meals to people who were not in a position to cook or care for themselves properly, such as for reasons of infirmity, mental or physical disability. Indeed, providing nutritious, balanced and affordable food, and also the friendship and human contact provided by the carers were seen as part of an important part of the healing process, particularly for those who had just left hospital.

Some of the social enterprises (Foxtrot, Hotel and India) reported that an outcome of enhancing people's knowledge and skills led to improvements in healthy behaviours and a decrease in illicit or self-destructive behaviours, particularly for those recovering from addictions to alcohol or drugs. Improvements in physical well-being and healing were also recognised by over half of the social enterprises (Alpha, Bravo, Echo, Golf and Hotel) as a consequence of bringing people together, enhancing their social contacts and building or widening people's social networks. Such personal contact was described as a ‘safety net’ for vulnerable people living in isolation:

“There's also that safety net that people are getting at, human contact, it might be the only one they get that day … On several occasions we've come back and said Mrs so and so's door, and she didn't answer … on several occasions the person has been lying behind the door like that, on at least five occasions that I know of …

She felt well if I stay in hospital I am going to die, so having gone home to her own house to be able to recuperate, to know there was that safety net there and all the rest of it, that helped her and did assist in her recuperation, so she's back, everything familiar all of that, so that's a massive difference to that lady.” (Alan)

The concept of the safety net is an important one in public health (Bartley et al., 1997) but has been employed particularly successfully in mental health (Ford, 1995, McCabe and Macnee, 2002), which is the focus of the next section.

#### 3.4.2 Mental health

The range of outcomes classified as relating to mental health include: improved confidence; improved coping and resilience; improved feelings of empowerment; increased sense of purpose and meaning; and improved sense of personal pride, dignity and self-worth.

Many interviewees reported there were improvements in confidence as a consequence of their activities, particularly as a result of enhancing people's knowledge and skills. This was the case whether it was through exposure to the arts, or through education and training programmes, such as those provided by several of the organisations involved in community development, or the ‘on the job’ training, as was often provided by the Work Integration Social Enterprises. Improvements in confidence were also seen as a consequence of working to improve people's social networks and bringing people together. Improved coping and resilience was also seen by two of the social enterprises (Juliet and Kilo) as being a direct consequence of providing access to quality information, such as welfare advice, and good advice and support was especially important for those seeking refuge or asylum (Bill).

Several social enterprises (Charlie, Delta, Foxtrot, Golf and India) reported improved feelings of empowerment in the individuals they supported as a consequence of their activities, including working to enhance knowledge and skills (such as through the arts), or through the meaningful work provided by the Work Integration Social Enterprises. A few of the social enterprises (Delta, Juliet and Kilo) reported that the beneficiaries of their activities gained an improved ‘sense of purpose’ and meaning in life, as a consequence of improving people's knowledge and skills and/or providing them with meaningful activity in a safe and supportive environment. People were perceived to have an improved sense of personal pride, dignity and self-worth as a result of their interaction with social enterprises (Bravo, Charlie and Foxtrot) and this was achieved in several different ways. Firstly, as a consequence of increased social interaction, and through the developing of new networks of friends; and secondly as a consequence of working to support vulnerable marginalised groups, such as homeless people (Charlie) or asylum seekers and refugees (Bravo), to feel that they are capable, productive members of society.

#### 3.4.3 Social determinants

Finally, a range of reported outcomes relate to known social determinants of health. These include: improved social capital, sense of community, feelings of trust and safety; sustained employment, increased income, enhanced future employability; and reduced stigmatization and marginalisation. As before, each outcome is discussed in turn.

Improving social capital, an increased sense of community and feelings of trust and safety were reported by most of the social enterprises (Alpha, Bravo, Delta, Echo, Hotel, Julie and Kilo) particularly as a consequence of bringing people together. Several of the social enterprises (Charlie, Delta, Hotel and India) reported that the people they had supported benefitted from sustained employment, increased income and enhanced future employability prospects. This was a consequence of supporting the people to improve their knowledge and skills, workers feeling that they had a supportive work environment, and also a consequence of improving access to information, particularly in relation to welfare. Indeed, four of the social enterprises (Bravo, Charlie, Foxtrot and India) reported a reduction in people feeling stigmatized or marginalised because of the support of the social enterprise. For example:

“That young person took part in stilt walking workshops and responded so well and what was positive from that very first encounter was suddenly all of the staff in the school who had great difficulty working with that young person suddenly saw her in a positive light … she was able to communicate with her peers in a way that she hadn't before because she was able to do this skill really quickly and really easily and obviously that was really cool that she could do that. She was able to communicate in a positive way with her teachers.” (Fiona)

Increased social contact and building friendships supported this process, but it was also a consequence of working to improve public awareness and understanding of particular social issues. Supporting marginalised people to feel that they are capable and contributing to society, such as through the work of Charlie in helping homeless people to earn their own income and ‘be their own boss’ can lead to reduced feelings of stigmatization:

“… they could beg and get money, but from selling the [magazine] it's not just someone dropping a coin in a cup or whatever, they actually have an interaction with a member of the public that they wouldn't normally get a chance to talk to … And the idea is that it empowers the person to kind of join back to society in whatever and being empowered into that by buying the street paper and selling it on the street … Once you've lost the feeling that you're responsible for your own self, it's pretty destructive …” (Charlie)

Social enterprises are increasingly seen as an important response to the marginalisation of individuals who experience challenges to full social participation: such work serves to “break down barriers and reduce stigma” (Lysaght et al., 2012, p. 455).

### 3.5 Presentation of the conceptual model

**Fig. 1:**
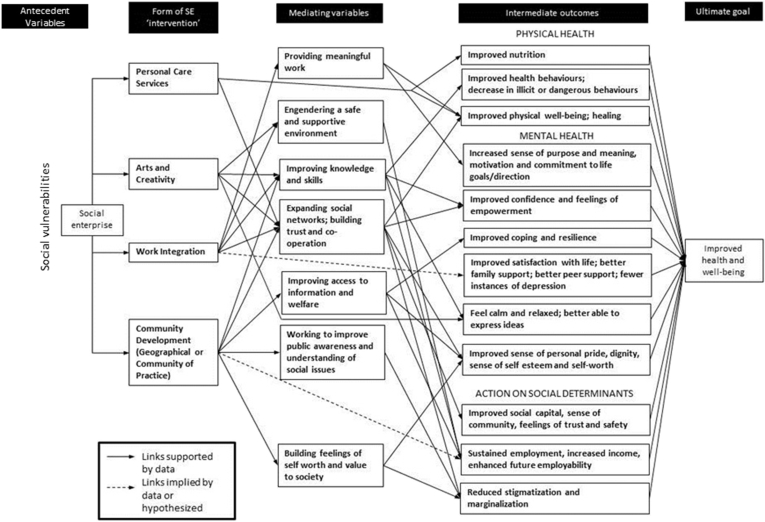
‘Empirically informed’ conceptual model of social enterprise as a health and well-being intervention.

The aim of the study was to develop an empirically informed conceptual model from the perspectives of those involved with running different social enterprises engaged in different types of activity. Through adopting a ‘systematic combining’ approach to theory building, drawing upon the analysis of the interview data, and building upon what was previously found from the systematic review of the literature (Roy et al., 2014), a model was prepared and an early version of this was presented at the focus group. Although broadly in agreement that the model was a fair distillation of their work, the participants raised several questions and provided several new insights which necessitated returning to the literature and data. For example, the importance of engendering a safe and supportive environment for people in the context of arts and creativity social enterprises was not something that had been picked up previously. However, after this point was raised in the focus group, evidence for this link was sought and subsequently found in the data and literature. Eventually enough links were mapped to enable the construction of the model shown at Fig. 1.

The model should be understood, first of all, to show that there is a range of highly context-specific antecedent variables relating to various aspects of social vulnerability. The social enterprise works to address these vulnerabilities in various ways, perhaps – although not always – employing particular organisational or legal forms related to the form of ‘intervention’ (for example, Work Integration Social Enterprises often taken the legal form of social co-operative in countries such as Italy). Although four different forms of ‘intervention’ have been identified during the course of this study (delivering personal care services, work integration, community development, and arts and creativity) these are not intended to be exhaustive by any means. As more studies are undertaken, more forms of intervention will be identified. The mediating variables column represents the wide array of ways in which social enterprises work to impact upon people's lives. We see that these are not specific to the type of intervention. We can also see a range of intermediate outcomes that have been grouped into three categories: physical health, mental health, and the social determinants of health. A wealth of existing literature can be drawn upon to connect each of the intermediate outcomes with the ‘ultimate goal’ of improved health and well-being. We can also see that two links (represented by a broken line) are only able to be implied or hypothesized at present, where evidence, particularly from the systematic review, was considered of insufficient quality to support the claims.

## 4 Discussion and conclusions

This paper sought to explore how social enterprise practitioners, implicitly or explicitly, conceptualise the impact of their activities upon the health and well-being of the individuals and communities they seek to support. It has been shown that social enterprises may impact upon physical health, mental health and/or the social determinants of health whether or not they explicitly *intend* to do so.

From the clustering of pathways relating to the mediating variable labelled ‘expanding social networks; building trust and co-operation’ in Fig. 1 we can infer the relative importance of this form of activity from the perspective of health and well-being, deserving of further exploration in future studies. The importance of social enterprise to building and enhancing social connectedness is recognised in an ever-growing number of studies (see, for example, Bertotti et al., 2012, Campbell and Sacchetti, 2014, Kay, 2006).

The consequence of recognising the public health role of actors operating outside of formal health systems could raise issues in relation to policy, particularly in relation to the allocation of resources. A case could potentially start to build for social enterprise and other third sector entities to be formally recognised, with a consequential ‘call’ on public health resources. But what would be the consequences when the role of the third sector starts to become recognised by healthcare systems? How, and in what circumstances, could that happen? By becoming part of the formal health services architecture, might they be encouraged to ‘scale up’ their activities to ‘fit’? What would be the consequences of this? Might this put their independence at risk? Or, through growth, leave the communities they were set up to support behind? (so called ‘mission drift’ – see Cornforth, 2014). We may be able to predict some of the issues that may arise by drawing upon literature from other fields that have studied the ‘creep’ of corporate practices and values (including professionalisation, marketisation and managerialism) from the for-profit sector, through the public sector and into the non-profit sector (Eikenberry and Kluver, 2004, Irvine, 2007).

An empirically informed conceptual model of causal assumptions linking the activities, intermediate outcomes, and ultimate goals together has been developed and presented, with a view to informing a future research agenda. There is considerable value in developing theoretical/conceptual models of implementation processes, particularly for using these theories as frameworks to shape and improve subsequent work, including evaluations (Chen and Rossi, 1983). One potential of the model relates to how it may be extended or pulled apart, with a focus, say, on different pathways or types of social enterprise, which can then be tested in different contexts.

There are, of course, inherent limitations in presenting models such as this, including some risks: the risk of obfuscating or ignoring the broader *structures* that mould and direct our everyday lives, for example. Nancy Krieger (2008, p. 223) reminds us that our “understanding of the societal distributions of health … cannot be divorced from considerations of political economy and political ecology”. Whether there is something implicit within social enterprises that enables them, or at least makes them potentially suitable, to address such structural factors, or whether their impact is constrained to dealing with (downstream) *symptoms* of problems alone, is a topic that deserves much closer critical examination (although see Roy (2016) and particularly Roy and Hackett (2016) for a full discussion). Glasgow also has a very specific health profile, and highly developed social enterprise sector. However, the use of purposive sampling and the provision of contextual information and ‘thick’ descriptions of findings with verbatim quotes, enables the reader to make their own assessment regarding the transferability of the claims made in this study to other contexts or settings (Koch, 2006, Lincoln and Guba, 2000).

As well as providing a platform for future research enquiry, it is hoped that the findings and conceptual work presented in this paper will contribute to contemporary debates in public health, particularly by encouraging a broader and more imaginative consideration of what actually constitutes a public health ‘intervention’. The claims made in this study are not intended to be the ‘truth’ by any means, rather an attempt to come up with a plausible model of how social enterprises *may have* a positive impact upon the health and well-being of individuals and communities and, if so, *how* and in *what ways*. It is intended that the conceptual models constructed represent a starting point, and will be subjected to further empirical testing, employing a range of methods, including both qualitative and quantitative in nature, and also more longitudinal case-study methods. In particular, future research would study the experiences of the ‘beneficiaries’ of social enterprises.

‘Developed’ nations spend an enormous – and ever growing – amount on healthcare. As demography changes and our society ages, difficult decisions will have to be made considering prioritisation of ever-scarcer resources. If we are truly interested in finding more efficient and effective ways to deal with complex, multi-faceted public health problems, including working to address health inequalities, then it should be a matter of public priority to examine the roles of a wide variety of actors, both within and outside of formal health systems. If it can be shown that investing in the work of the third sector, rather than in more conventional public health services will yield ‘better’ results (howsoever determined) in the long term, and perhaps avoid – or at least lessen – the diminishing returns that we currently see from investment in healthcare, then this may prove to be a significant advance.

## Figures and Tables

**Table 1:** Characteristics of the sampled social enterprises.

Social enterprise	Respondent (sex)	Brief description	Reach	Turnover (in GBP) (as at EOY, 2013)	Employees (FTEs) (at EOY, 2013)
Alpha	Alan (M)	Provides a range of personal services, such as prepared meal deliveries (‘meals on wheels’) and other services such as cleaning and ironing to elderly, sick and disabled people who, for reasons of disability or illness, cannot cope on their own.	Local	£134,000	3
Bravo	Bill (M)	Former ‘healthy living centre’, provides a range of services, including a range of advice and advocacy services for local asylum seekers and refugees; a counselling service; credit union; ESOL classes; drop-in centre; education and cultural services provision.	Local	£211,815	3
Charlie	Christine (F)	Supports the establishment and development of organisations, on a worldwide basis, which publish and supply street papers and magazines to help homeless and/or unemployed people to gain income.	International	£383,366	7
Delta	Doreen (F)	Community centre providing a range of services and facilities for local people, particularly those who are unemployed, to enhance their education and employability skills.	Local	£221,575	4.5
Echo	Edward (M)	Provides a range of care services for people with significant support needs, including people with severe mental and physical disabilities.	City	£510,000	9.5
Foxtrot	Fiona (F)	Works with young people, particularly of school age at risk of exclusion, and trains them in circus skills.	National	£200,000	5
Golf	Gail (F)	Supports offenders soon to be released from prison, or soon after release, through the arts (such as music, art, theatre and drama) to ease their transition to the outside world and help prevent re-offending.	National	£111,997	2
Hotel	Harry (M)	Trains and employs people to collect and receive donations of old and scrap wood from all over the city, mainly for ‘up-cycling’ into useful products to sell.	City	£284,293	5.5
India	Ian (M)	Provides training, work experience, guidance and support, personal development, education and social activities for young people and adults experiencing disability and/or social disadvantage through the provision of a range of services, including providing catering and café facilities.	National	£2,493,000	71
Juliet	Jill (F)	Former ‘healthy living centre’ provides a wide range of projects and services aimed at supporting local people with poor mental and physical health; elderly people; isolated people; carers and their families; and local organisations who need help to expand.	Local	£391,152	7.5
Kilo	Karen (F)	Provides common area cleaning and property repair and maintenance services to the local housing association and to other social landlords, allowing them to employ and train local unemployed young people, operate an extensive apprenticeship programme, and reinvest their profits into a range of activities aimed at improving the lives of people in the local community.	Local	£1,298,353	23
Lima	Laurence (M)	Aims to develop a range of projects focused upon environmental protection of the local area, improvement of public open space and a range of environmental and regeneration projects, including local amenities.	Local	£33,557	1.5
Mike	Martin (M)	Preservation of local historical, architectural and constructional heritage. Provides a range of services to the public, including the employment and training of people in a range of environmental projects, and a community café selling and utilising produce grown on the site.	Local	£469,097	11
